# *Solanum lyratum*-Derived Solalyraine A1 Suppresses Non-Small Cell Lung Cancer Through Regulation of Exosome Secretion and Related Protein Biomarkers

**DOI:** 10.3390/ph18091280

**Published:** 2025-08-27

**Authors:** Pu Jiang, Liangyu Liu, Lixian Chen, Bing Han, Xiao Du

**Affiliations:** 1 School of Pharmacy, Hangzhou Normal University, Hangzhou 311121, China; jpaihb@163.com; 2 Jinfeng Laboratory, Yu-Yue Pathology Scientific Research Center, Chongqing 400039, China; 3 State Key Laboratory of Natural and Biomimetic Drugs, School of Pharmaceutical Sciences, Peking University, Beijing 100091, China; liuliangyu93@126.com (L.L.); 2010307105@stu.pku.edu.cn (L.C.); 4 School of Pharmaceutical Sciences, Zhejiang Chinese Medical University, Hangzhou 310053, China

**Keywords:** *Solanum lyratum*, steroidal glycoalkaloid, solalyraine A1, NSCLC, exosome, proteomics

## Abstract

**Background**: Lung cancer is a prevalent malignancy globally, with non-small cell lung cancer (NSCLC) accounting for 80–85% of cases. Solalyraine A1 (SA1) is a steroidal glycoalkaloid derived from *Solanum lyratum*. However, the effect and mechanism of SA1 on NSCLC remain unclear. **Methods**: The exosomes from SA1-treated A549 cells were prepared and administered to A549 xenograft mice. Proteomics analysis of SA1-treated A549 cells and their exosomes was conducted to assess the mechanism. Bioinformatics analysis was utilized to identify differentially expressed proteins (DEPs) and key signaling pathways. Western blot analysis confirmed the expression of potential targets. **Results**: SA1 effectively suppressed tumor growth in A549 xenografts, demonstrating a remarkable inhibition rate of 70.48%. A total of 1154 DEPs were identified in A549 cells, primarily associated with the ribosome pathway. Additionally, 746 DEPs were identified in exosomes, mainly involved in the spliceosome pathway. Five highly regulated DEPs were selected for verification. SA1 was found to suppress MUC5B and elevate APOB expression in A549 cells, while inhibiting MFGM, ANGL4 and increasing GCN1 expression in exosomes. **Conclusions**: This study demonstrates that SA1 exhibits anti-NSCLC effects by regulating exosome function and related protein expression, providing novel insights for NSCLC treatment.

## 1. Introduction

Lung cancer remains the primary cause of cancer-related mortality, with 2.2 million new cases and 1.8 million deaths reported in 2020. Non-small cell lung cancer (NSCLC) constitutes 80% to 85% of all lung cancer cases [1]. Since most patients have entered the advanced stage at the time of diagnosis, conventional treatments like surgery, radiotherapy, and chemotherapy offer limited clinical benefits, resulting in a 5-year survival rate below 17% [2]. While molecular targeted therapies have shown promise, they are only effective for a subset of patients with specific gene mutations, and eventual development of acquired resistance is common [3]. Hence, there is an urgent need to develop novel anti-NSCLC drugs and identify new therapeutic targets for this disease.

**Figure 1:**
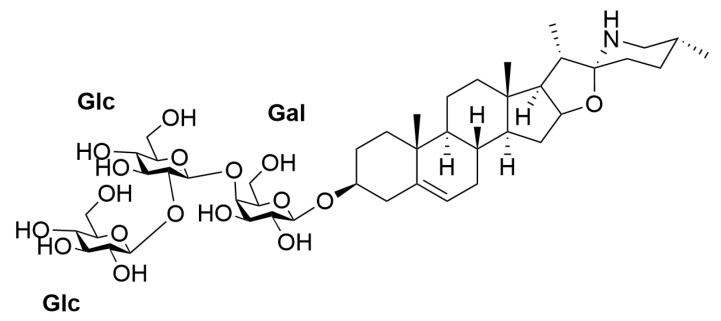
The structure of SA1.

*Solanum lyratum* Thunb., a plant belonging to the Solanaceae family, was documented in China’s earliest extant pharmacological monograph “Shen Nong’s Herbal Classic”, where it was classified as a top-grade herbal medicine with no recorded toxicity. It has been widely used in traditional Chinese medicine for the treatment of various cancers through long-term clinical applications [4]. The excellent anti-cancer effect of *S. lyratum* has attracted considerable attention. The alkaloids derived from this plant have been identified as the major anti-tumor bioactive components [5]. In our previous study, we reported that the total steroidal glycoalkaloids from *S. lyratum* (TSGS) exhibit remarkable anti-NSCLC activity, with an IC_50_ value of 93 μg/mL in A549 cells [6] and an inhibition rate of 68.43% in A549 xenograft model at a dose of 200 mg/kg [7]. In addition, solalyraine A1 (SA1) (Figure 1), a specific steroidal glycoalkaloid isolated from TSGS, has been shown to significantly suppress cancer cell migration, invasion, and tube formation in tumor-derived vascular endothelial cells [8,9]. Furthermore, we demonstrated that SA1 could inhibit the pro-angiogenic activity of A549-derived exosomes (ADEs) [10]. However, the underlying mechanism through which SA1 exerts its inhibitory effects on NSCLC remains to be elucidated.

**Figure 2:**
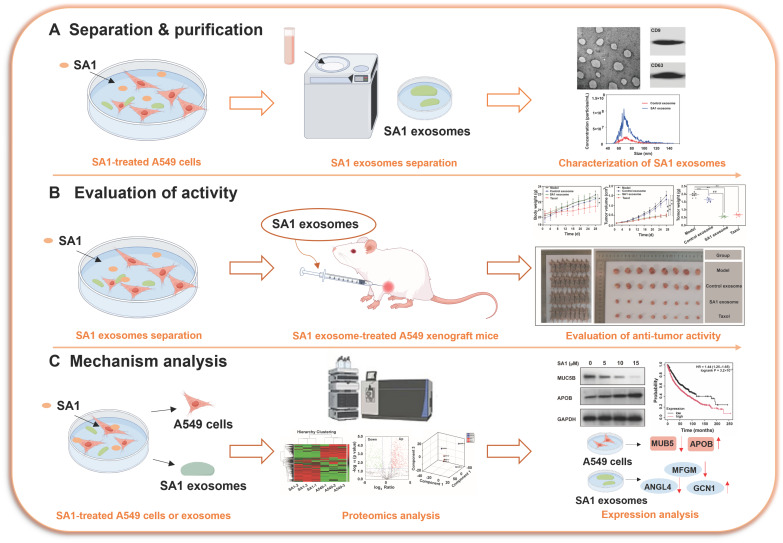
The research schematic diagram. (**A**) Preparation and characterization of exosomes from SA1-treated A549 cells. (**B**) Assessment of the anti-tumor efficacy of SA1 exosomes in A549 xenograft mice. ** *p* < 0.01, compared with model group; ## *p* < 0.01, compared with control exosome group. (**C**) Proteomics analysis and validation of key proteins after SA1 treatment.

Exosomes are a kind of 30–150 nm vesicles released by most type of cells including tumor cells. Emerging evidence indicates that tumor-derived exosomes (TDEs) facilitate tumor metastasis by transferring bioactive molecules to establish the premetastatic niche [11]. In addition, TDEs contribute to tumor angiogenesis, drug resistance, and immune evasion [12]. Thus, the molecular cargo of TDEs warrants detailed investigation, and proteomic profiling of TDEs may reveal potential anti-cancer targets. On the other hand, as the exosomes originate from the membrane lipid rafts, their formation is highly reliant on lipid rafts and cholesterol on the cell membrane [13]. Steroidal glycoalkaloids share structural similarities with certain steroidal saponins, which can disrupt membrane integrity by forming molecular complexes with membrane cholesterol. We thus speculated that SA1, a steroidal glycoalkaloid, may inhibit tumor progression by binding to membrane cholesterol and perturbing lipid raft organization, ultimately affecting TDE function. Therefore, in this study, SA1 exosomes was administered to A549 tumor xenograft mice in vivo. A label-free quantitative method was performed to analyze the proteomes of both A549 cells and their derived exosomes. Subsequent bioinformatics analyses were conducted to identify key regulatory pathways, and Western blotting was used to validate the proteomic findings (Figure 2).

## 2. Results

### 2.1. Characterization of Exosomes

**Figure 3:**
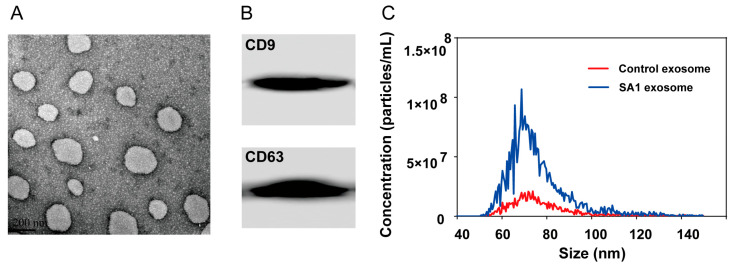
The characterization of exosomes. (**A**) Transmission electron microscopy image. (**B**) Western blot images of exosomal protein markers CD9 and CD63. (**C**) Nanoparticle tracking analysis determined the size of exosomes.

The ADEs were successfully isolated from the A549 cell medium, as verified by transmission electron microscopy (Figure 3A), the expression of exosomal protein markers (CD9 and CD63) in the resulting exosomes (Figure 3B), and nanoparticle tracking analysis to detect the size and quantity of the vesicles. After SA1 treatment, the particle size was found to be 75.90 ± 14.14 nm and the concentration was found to be 3.34 × 10^9^/mL (Figure 3C and Table 1).

### 2.2. Exosomes Derived from SA1-Treated A549 Cells Inhibits NSCLC Tumor Growth

**Figure 4:**
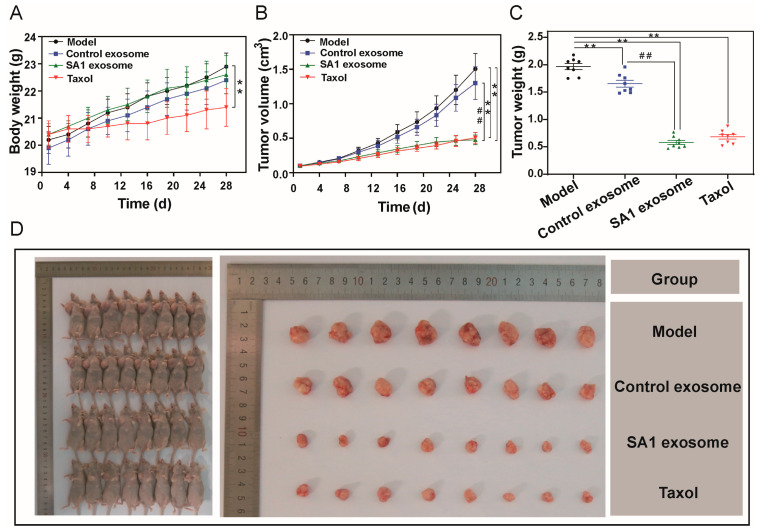
Effects of SA1 on tumor growth in A549 xenograft mice. (**A**) Body weight. (**B**) Tumor volume. (**C**) Tumor weight of mice recorded at the end of the study. (**D**) Images of the sacrificed A549 xenograft mice and excised tumors. Values are presented as mean ± SD, *n* = 8. ** *p* < 0.01, compared with model group; ## *p* < 0.01, compared with control exosome group.

No apparent changes were observed in the appearance or behavioral status of the mice throughout the experiment. The body weight in the model group, control exosome group, and SA1 exosome group showed a considerable increasing trend, whereas a more moderate increase was observed in the Taxol group (Figure 4A). Exosomes derived from SA1-treated cells markedly inhibited tumor growth in A549 xenograft mice. As shown in Figure 4B,D, administration of SA1 exosomes led to a marked reduction in tumor volume from 1506 ± 223 mm^3^ in the model group to 478 ± 7 mm^3^ in the SA1 exosome group, and reduced tumor weight from 1.97 ± 0.16 g to 0.58 ± 0.11 g. Compared with the model group, significant suppression of tumor growth was observed in the SA1 exosome group, control exosome group, and Taxol group. The control exosome group showed a modest tumor inhibition rate of 15.94%. Notably, the SA1 exosome group demonstrated the highest tumor inhibition rate of 70.48%, surpassing even that of Taxol group (65.21%), indicating a potent anti-NSCLC effect. The tumor inhibition rates for all groups are summarized in Table 2.

### 2.3. Global Proteomics Analysis of SA1-Treated A549 Cells and the Derived Exosomes

**Figure 5:**
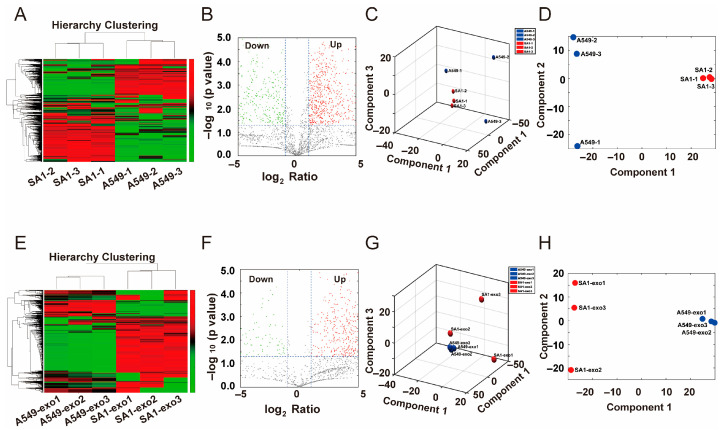
Global proteomics analysis of SA1-treated A549 cells and the secreted exosomes. (**A**) Hierarchy clustering, (**B**) volcano plot, (**C**) PCA-3D, and (**D**) PCA-2D of DEPs from SA1-treated A549 cells vs. A549 cells. (**E**) Hierarchy clustering, (**F**) volcano plot, (**G**) PCA-3D, and (**H**) PCA-2D of DEPs in exosomes from SA1-treated A549 cells vs. A549 cells.

To elucidate the mechanism underlying SA1-mediated tumor suppression, we conducted label-free quantitative proteomics analysis on both SA1-treated A549 cells and their secreted exosomes. In SA1-treated A549 cells, 1154 differentially expressed proteins (DEPs) were identified, including 692 upregulated and 462 downregulated proteins compared to the control group (Figure 5A,B). The samples within each group exhibited high reproducibility, and a clear separation was observed between SA1-treated and control cells (Figure 5C,D). In the ADE groups, 746 DEPs were detected upon SA1 treatment, with 629 upregulated and 117 downregulated proteins relative to the control group (Figure 5E,F). The PCA further revealed distinct clustering between exosomes from A549 vs. SA1-treated A549 cells (Figure 5G,H). These results indicated that SA1 treatment significantly altered the proteomic profiles of both A549 cells and their secreted exosomes.

### 2.4. Protein Enrichment and Function Analysis

**Figure 6:**
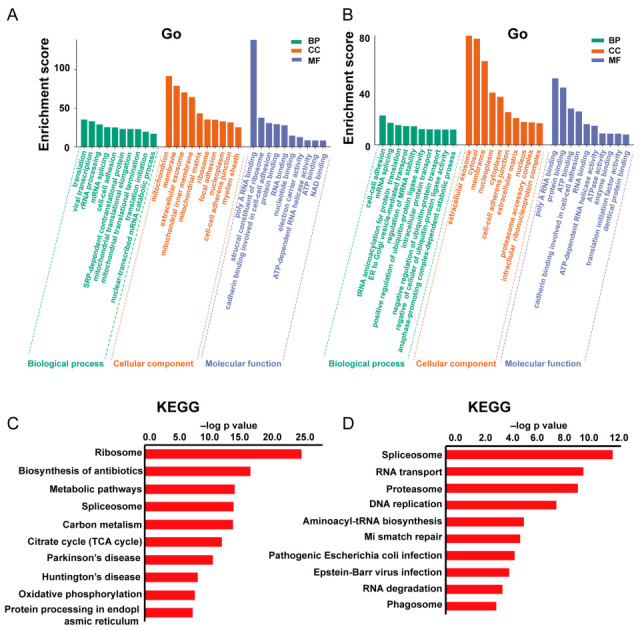
GO and KEGG analysis of DEPs. (**A**) GO and (**C**) KEGG analysis of DEPs from A549 cells vs. SA1-treated A549 cells. (**B**) GO and (**D**) KEGG analysis of DEPs from exosomes of A549 cells vs. SA1-treated A549 cells.

**Figure 7:**
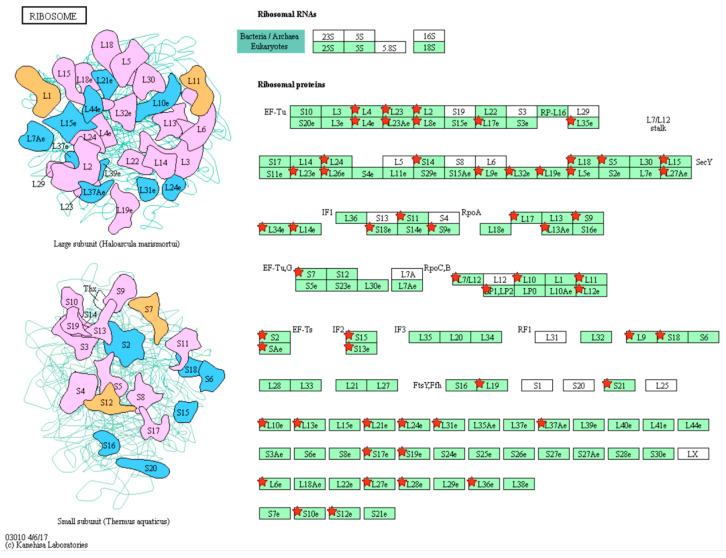
Ribosome pathway and the identified proteins in A549 cells. Red star indicated the DEPs identified in this study.

**Figure 8:**
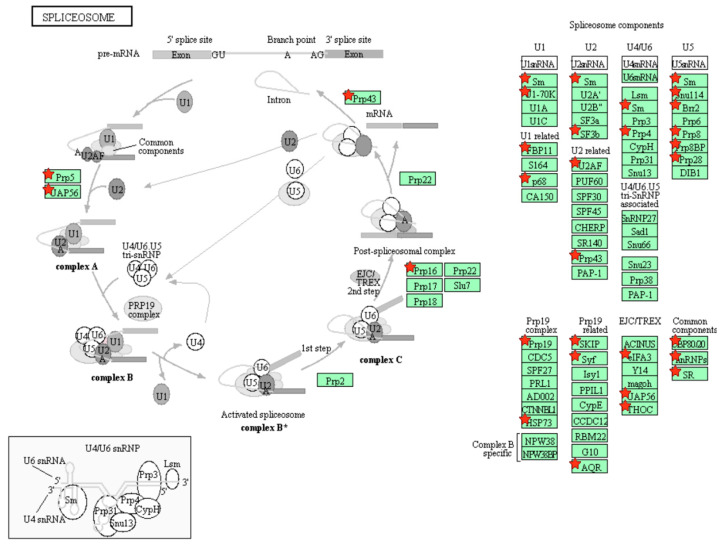
Spliceosome pathway and the identified proteins in ADEs. Red star indicated the DEPs identified in this study.

Gene ontology (GO) enrichment analysis was first conducted to characterize the DEPs. The top ten significantly enriched GO terms are shown in Figure 6A,B. In SA1-treated A549 cells, the DEPs were predominantly located in mitochondria, cell membrane, and extracellular exosomes. Their molecular functions were primarily associated with poly(A) RNA binding, and they were involved in biological processes including translation, rRNA processing, mRNA splicing, cell–cell adhesion, and mitochondrial translation (Figure 6A). For DEPs derived from exosomes, major localization included extracellular exosomes, cytosol, and membranes. Key molecular functions encompassed poly(A) RNA binding, protein binding, and ATP binding, with significant involvement in biological processes such as cell–cell adhesion, mRNA splicing, and tRNA aminoacylation for protein translation (Figure 6B). Subsequent Kyoto encyclopedia of genes and genomes (KEGG) enrichment analysis showed that the ribosome, biosynthesis of antibiotics, metabolic pathways, and spliceosome were significantly involved after SA1 treatment (Figure 6C). In exosomes from SA1-treated A549 cells, the most enriched pathways included the spliceosome, RNA transport, proteasome, and DNA repair (Figure 6D). The top enriched pathway and corresponding proteins are shown in Figure 7 and Figure 8.

**Figure 9:**
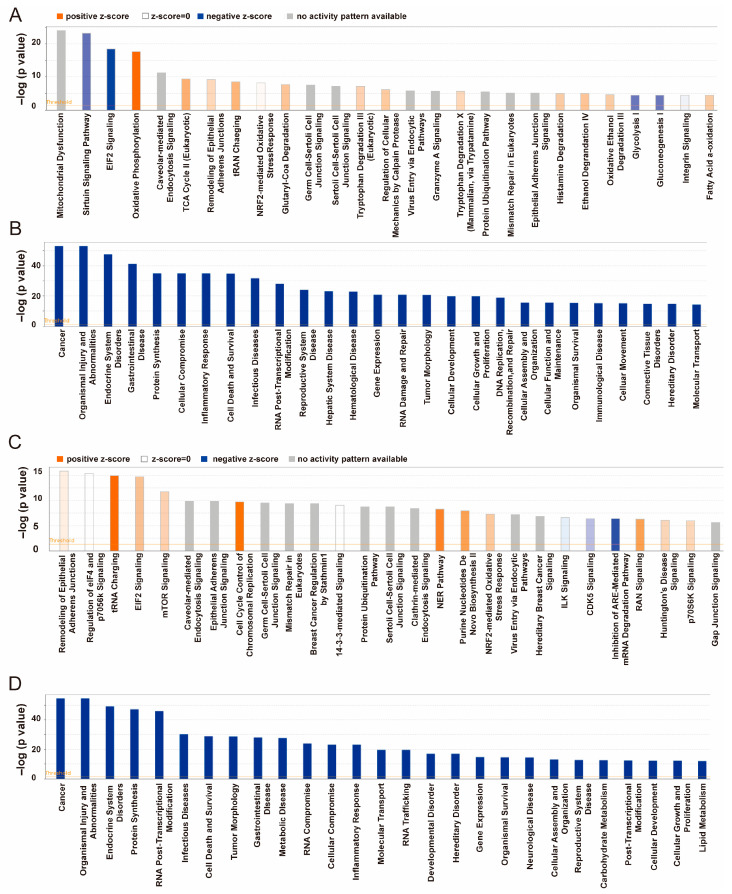
Ingenuity pathway and function enrichment analysis of DEPs. (**A**,**B**) Pathway and function enrichment analysis of DEPs from A549 cells vs. SA1-treated A549 cells. (**C**,**D**) Pathway and function enrichment analysis of DEPs in exosomes from A549 cells vs. SA1-treated A549 cells.

Ingenuity pathway analysis identified 534 signaling pathways related to DEPs in A549 cells. The signaling pathways ranked by significance are shown in Figure 9A, among which the most significantly enriched pathways were mitochondrial dysfunction, sirtuin signaling, EIF2 signaling, and oxidative phosphorylation pathways (Figure 9A). For exosomal DEPs, 499 pathways were identified, with the top pathways being remodeling of epithelial adherent junctions, regulation of eIF4 and p70S6K signaling, tRNA charging, EIF2 signaling, and mTOR signaling pathways (Figure 9C). Function enrichment analysis indicated that SA1 treatment regulated processes related to cancer, protein synthesis, RNA post-transcriptional modification, cell death and survival, and tumor morphology (Figure 9B). Similarly, exosomal DEPs were enriched in functions associated with cancer, protein synthesis, cell death and survival, RNA post-transcriptional modification, tumor morphology, and cellular growth and proliferation (Figure 9D).

### 2.5. Validation of Significantly Regulated DEPs

**Figure 10:**
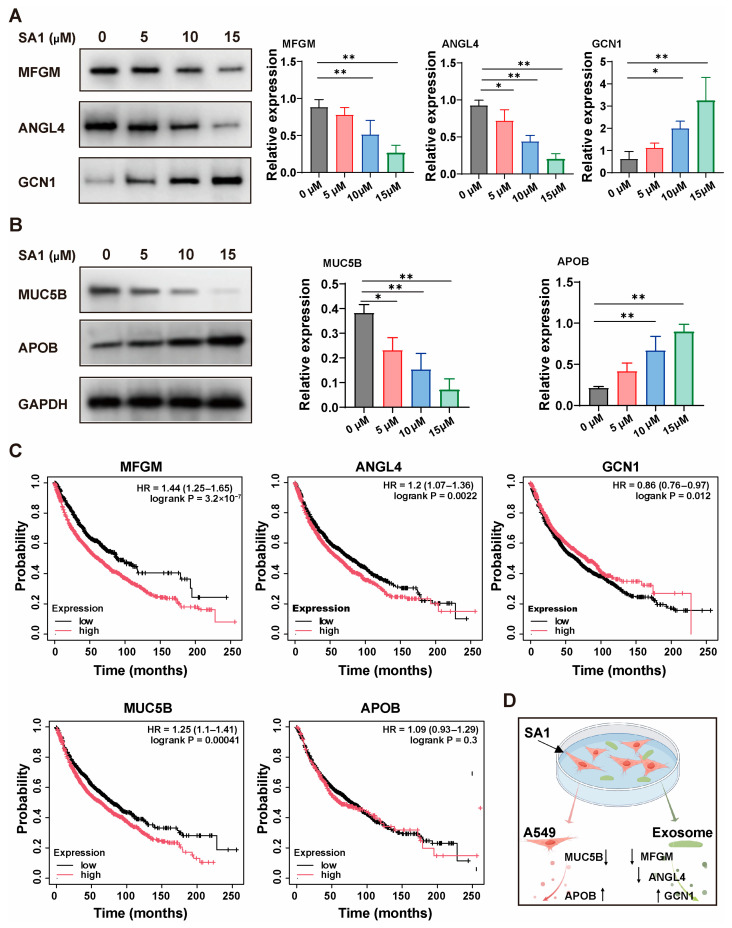
Verification of key DEPs in A549 cells and the exosomes. (**A**) The relative expression of MFGM, ANGL4, and GCN1 in exosomes from SA1-treated A549 cells. The protein expression was normalized by the protein level in the control. (**B**) The relative expression of MUC5B and APOB in A549 cells after SA1 treatment. The protein expression was normalized by GAPDH. Data were expressed as the mean ± SD, *n* = 3. * *p* < 0.05 and ** *p* < 0.01 compared with control group. (**C**) The overall survival analysis of MFGM, ANGL4, GCN1, MUC5B, and APOB in lung cancer patients using Kaplan–Meier Plotter database. (**D**) The schematic diagram of the mechanism.

To validate the proteomic findings, we selected five highly regulated DEPs, including Mucin-5B (MUC5B) and Apolipoprotein B (APOB) in A549 cells (Table 3) and Lactadherin (MFGM), Angiopoietin-related protein 4 (ANGL4), and Stalled ribosome sensor GCN1 (GCN1) in exosomes, for further confirmation by Western blot (Table 4). The results demonstrated that SA1 significantly upregulated APOB expression in A549 cells and GCN1 in exosomes, while downregulating MUC5B in cells, as well as MFGM and ANGL4 in exosomes (Figure 10A,B), which was consistent with the proteomics data. Bioinformatic analysis revealed that high expression of MFGM (logrank *p* = 3.2 × 10^−7^), ANGL4 (logrank *p* = 0.0022), and MUC5B (logrank *p* = 0.00041) was associated with poor overall survival in lung cancer patients, whereas elevated GCN1 levels (logrank *p* = 0.012) correlated with better survival. In contrast, APOB expression (logrank *p* = 0.3) showed no significant association with patient survival (Figure 10C). These findings suggest that MUC5B, MFGM, ANGL4, GCN1, and APOB may play critical roles in mediating the anti-NSCLC effects of SA1 (Figure 10D).

## 3. Discussion

With the rising morbidity and mortality of lung cancer, effective strategies for preventing and treating NSCLC remain challenging. In this study, SA1 was investigated as a potential anti-NSCLC agent. The results demonstrated that exosomes derived from SA1-treated A549 cells significantly suppressed tumor growth in A549 xenograft mice. Notably, the tumor inhibition rate in the SA1 exosome group exceeded that of the Taxol group, a commonly used first-line clinical chemotherapeutic agent. Given that TDEs promote tumor survival and progression by facilitating intercellular communication, targeting TDE formation represents a promising therapeutic strategy. However, the control exosome group also showed an inhibitory effect, which appears inconsistent with the generally recognized pro-tumor role of TDEs. One possible explanation is that when numerous exosomes were injected into mice, the immune responses in the body might have been strongly stimulated to fight against them, thereby enhancing anti-tumor immunity. Nevertheless, the underlying mechanisms need to be better understood and exploited. Despite this observation, the findings still strongly suggest that SA1 exhibits significant anti-NSCLC property by intervening ADEs.

TDE vesicles originate from the outward budding of lipid rafts in the plasma membrane, implicating lipid rafts not only in TDE biogenesis but also in their mechanisms of action on target cells. Beyond providing structural integrity, lipids play an active role in exosome formation [14]. Similarly to steroidal saponins, steroidal glycoalkaloids can form a molecular complex precipitated with cholesterol and directly agglutinate cholesterol of lipid rafts [15]. Our data indicates that SA1 intervention might increase the number of exosomes secreted by A549 cells. As a steroidal glycoalkaloid, SA1 is speculated to mediate its effects on ADEs by agglomerating cholesterol within the tumor cell membrane. This process likely affects lipid raft integrity, thereby impairing exosome formation and altering exosomal functions. This hypothesis is indirectly supported by previous findings [16]. Additionally, several top DEPs identified in the proteomic analysis are associated with lipid metabolism. For instance, ANGL4 inhibits lipoprotein lipase activity, thereby regulating triglyceride clearance and lipid metabolism [17]. APOB serves as a primary protein component of chylomicrons, low-density lipoprotein, and very-low-density lipoprotein [18]. Nevertheless, this hypothesis warrants further experimental validation.

The proteomics can help find the molecular mechanisms underlying cancer occurrence and identify cancer-related proteins, which are commonly used for cancer research [19]. In this study, the label-free quantitative method and bioinformatics were employed to analyze the proteomes of A549 cells and ADEs. Comparative analysis between the control and SA1 groups demonstrated a significant difference, which revealed potential anti-NSCLC mechanisms of SA1 and provided directions for further study. The most significant DEPs of cells and TDEs were followed with interest.

Compared with the control group, 692 DEPs of cells were upregulated after SA1 intervention, among which the most significant protein was APOB. As a key lipid transport protein, APOB may be involved in metabolic reprogramming of NSCLC cells. Previous studies have reported significantly abnormal APOB expression in plasma exosomes of lung cancer patients with liver metastasis, suggesting its potential as a novel biomarker for diagnosing lung cancer [20]. In addition, the genetic variant rs1801701 in APOB was significantly associated with overall survival in NSCLC patients [21]. Increased expression of the APOB mRNA editing catalytic subunit-like enzyme, APOBEC3B, has also been observed in NSCLC patients receiving EGFR-targeted therapy [22]. Our results demonstrate that SA1 significantly upregulates APOB expression, an effect consistent with that of paclitaxel. We propose two potential mechanisms: first, SA1 may bind to cholesterol, reducing cholesterol levels and preventing its binding to APOB, thereby sparing APOB from degradation and increasing its expression; second, SA1 may enhance APOB expression to inhibit cholesterol digestion and transport. Although the exact mechanisms remain unclear, this study confirms that SA1 can inhibit tumor progression by modulating APOB-related lipid metabolism processes, indicating that APOB is a promising new target worthy of further investigation.

Another significantly regulated DEP by SA1 is MUC5B. As a mucin, overexpression of MUC5B is closely associated with enhanced invasiveness and poor prognosis in multiple cancers [23]. A study based on the proteomic profiling of plasma exosomes from lung cancer patients with metastasis suggested that MUC5B could serve as a potential biomarker for diagnosing lung cancer brain metastasis [20]. Furthermore, the promoter polymorphism of MUC5B (rs35705950) was significantly correlated with worse overall survival in NSCLC patients receiving radiotherapy [24]. Another pathological analysis of NSCLC patients carrying EGFR mutations revealed that MUC5B expression levels in tumor tissues were strongly associated with patient overall survival, indicating that MUC5B may act as a novel prognostic biomarker in this subgroup [25]. Our results show that SA1 significantly downregulates MUC5B expression. Based on the aforementioned literature, we speculate that SA1 may inhibit malignant phenotypes such as tumor proliferation, metastasis, and invasion through suppressing MUC5B. However, the specific molecular mechanisms by which SA1 regulates MUC5B, and whether additional effector molecules are involved in this pathway, require further in-depth investigation.

Compared with the control group, 629 DEPs of exosomes were upregulated after SA1 intervention, among which the most significant regulated protein was GCN1, the activator of EIF2-alpha kinase GCN2. GCN1 functions as a ribosome collision sensor and plays a pivotal role in the RNF14–RNF25-mediated translation quality control pathway. This pathway is activated when ribosome stalling occurs during translation, leading to the ubiquitination and subsequent degradation of associated translation factors on the stalled ribosomes [26]. However, to the best of our knowledge, there are few studies on the relation of GCN1 with cancer except that GCN1 was reported to be overexpressed in prostate cancer [27]. Our results indicated that SA1 acted on the protein expression in exosomes and affected GCN1 function, but further molecular mechanisms still need to be explored.

Additionally, SA1 treatment also resulted in the downregulation of 117 exosomal proteins, with MFGM (also known as MFG-E8) being the most significantly suppressed. MFGM is an anti-inflammatory glycoprotein implicated in the regulation of multiple pathophysiological processes. Previous studies have reported that MFGM is overexpressed in various cancers and is considered a key regulator of cancer cell invasion, migration, and proliferation. For example, MFGM is an oncogenic protein in angiosarcoma and patients with overexpressed MFGM showed short progression-free survival and overall survival time [28]. In addition, MFGM is highly expressed and associated with poor prognosis in bladder urothelial carcinoma [29]. Also, MFGM expression is significantly higher in hepatocellular carcinoma than in normal liver tissues and contributes to the disease progression [30]. Our findings demonstrate that SA1 significantly inhibits MFGM expression in exosomes, suggesting that MFGM may serve as a critical target through which SA1 exerts its anti-tumor effects.

Another protein significantly regulated by SA1 is ANGL4, a secreted protein belonging to the angiopoietin-like family, which plays a key role in promoting angiogenesis and tumor metastasis. In NSCLC cells, ANGL4 is involved in regulating metabolic processes such as aerobic glycolysis, glutamine consumption, and fatty acid oxidation, thereby influencing cell proliferation and energy homeostasis [31]. It is worth noting that under hypoxic conditions, ANGL4 expression was significantly upregulated in NSCLC cells compared to the normoxic group, suggesting its responsiveness to low-oxygen signals in the tumor microenvironment. Furthermore, studies have shown that alterations in ANGL4 expression are positively correlated with radio-resistance in NSCLC cells and xenograft tumors, indicating its potential complex role in DNA damage response and radiotherapy resistance [32]. In this study, SA1 significantly inhibited the secretion of ANGL4 in exosomes. This effect may attenuate the protein’s cancer-promoting functions, thereby partially suppressing malignant tumor progression. These findings are consistent with multiple previous studies and further support the value of ANGL4 as a potential target for cancer therapy.

Following functional analysis of the aforementioned proteins, we identified that MFGM, APOB, and MUC5B are closely associated with “membrane structure” and “secretory processes”. Previous studies have indicated that increased membrane permeability under pathological conditions may lead to ion influx and leakage of intracellular components. Based on these findings, we hypothesize that SA1 may contribute to the maintenance of cellular lipid metabolic homeostasis and membrane stability through regulating MFGM, APOB, and MUC5B. It has been reported that homologs or structural analogs of SA1 are involved in lipid metabolism [33,34]. On the other hand, the expression of ANGPTL4 and GCN1 is closely linked to cellular stress and metabolic dysregulation. Within the tumor microenvironment, conditions such as hypoxia and nutrient deprivation can exacerbate cellular stress, leading to dysregulated expression of ANGPTL4 and GCN1. We further speculate that SA1 may modulate these proteins to help cells adapt to stress, thereby potentially playing a role in counteracting stress responses.

Based on the pathway and function enrichment results, we observed that the DEPs in A549 cells were predominantly localized to the mitochondria. Beyond their role in providing energy, mitochondria participate in the process of generating nucleic acid precursors, the basic unit of DNA. Without mitochondria, tumor cells are unable to synthesize new DNA and thus cannot proliferate [35]. Furthermore, the most significantly enriched signaling pathways associated with the DEPs included mitochondrial dysfunction and cancer-related pathways. It has been reported that mitochondrial dysfunction affects tumor cells directly or indirectly and targeting mitochondria has been proposed as a promising strategy for cancer therapy [36]. These findings suggest that SA1 may affect mitochondrial function by regulating mitochondrial proteins of A549 cells. Further mechanistic studies should therefore focus on mitochondrial regulation. Additionally, our results indicated that SA1 significantly affected proteins associated with exosomes, implying that SA1 may influence exosome formation or function. This observation further supports the relevance of exosomal proteome analysis in this context.

Apart from the mitochondrial dysfunction pathway, the DEPs in A549 cells were significantly enriched in the sirtuin pathway. The sirtuin protein family (SIRT1–SIRT7) plays crucial roles in tumorigenesis. SIRT1 is involved in DNA damage repair and contributes to genomic stability, thereby influencing tumor cell proliferation [37]. It also modulates the TGF-β signaling pathway and suppresses tumor metastasis [38]. SIRT3, SIRT4, and SIRT5 are mainly localized in mitochondria, where they regulate various mitochondrial functions. Moreover, SIRT3 and SIRT4 have been shown to inhibit tumor cell proliferation [39,40]. SIRT6 can regulate gene expression of DNA stability and immunity [41], while SIRT7 promotes tumor metastasis through upregulation of E-cadherin expression [42]. These findings suggest that SA1 may suppress tumor proliferation and metastasis by modulating the activity of sirtuin family proteins.

Ribosome biogenesis is frequently upregulated in tumors and contributes to the early stages of tumorigenesis. A hallmark of cancer cells is their enhanced ribosome function, which supports the increased protein synthesis required for rapid proliferation [43]. Studies have shown that ribosome inhibitors induce ribotoxic stress by activating signaling pathways such as JNK and p38, thereby promoting tumor cell apoptosis [44]. Additionally, cancer cells may exploit ribosomes to evade immune detection and facilitate immune escape [45]. The splicing of precursor mRNA (pre-mRNA) is catalyzed by a dynamic ribonucleoprotein complex known as the spliceosome. Although traditionally considered a housekeeping mechanism, mutations in core spliceosome components are frequently associated with tumorigenesis. Notably, spliceosome-targeted therapy has emerged as a promising anti-cancer strategy, particularly for cancers with spliceosome deficiencies [46]. For instance, the small-molecule spliceosome inhibitor E7107 has been shown to suppress drug resistance recurrence and malignant progression in prostate cancer [47]. Furthermore, targeting the spliceosome can lead to the accumulation of mis-spliced RNAs, which activate antiviral-like immune responses and trigger cell death in cancer cells [48]. Thus, both the ribosome and spliceosome represent valuable therapeutic targets in oncology. In this study, we demonstrated that SA1 treatment significantly modulates the ribosome pathway in NSCLC cells and the spliceosome pathway in ADEs. Based on these findings, we hypothesize that SA1 may disrupt normal ribosome function in A549 cells, thereby interfering with protein translation and synthesis. Some of the affected proteins may in turn regulate the expression of related genes. This ultimately leads to differences in the expression profiles of genes and proteins in exosomes. The pronounced alterations in related proteins suggest that these pathways may underlie the anti-NSCLC mechanisms of SA1. However, further experimental validation is required to confirm these findings.

## 4. Materials and Methods

### 4.1. Regents

SA1 was isolated from *S. lyratum* according to our previous study [8]. Taxol was purchased from MedChemExpress (No. HY-B0015, Shanghai, China). RPMI-1640 medium, fetal bovine serum, and penicillin/streptomycin were obtained from Gibco (Big Cabin, OK, USA). The following antibodies were used: CD9 (Proteintech, Wuhan, China, No. 20597-1-AP), CD63 (Proteintech, No. 25682-1-AP), MFGM (Proteintech, No. 67797-1-Ig), ANGL4 (Proteintech, No. 18374-1-AP), APOB (Proteintech, No. 20578-1-AP), GADPH (Proteintech, No. 10494-1-AP), MUC5B (Santa Cruz, Dallas, TX, USA, No. sc-21768), GCN1 (ABclonal, Woburn, MA, USA, No. A19851), and IgG-HRP (KeyGen Biotech, Nanjing, China, No. KGAA35).

### 4.2. Cell Line and Culture Conditions

The human lung cancer cell line A549 was obtained from Jiangsu KeyGen Biotech Co., Ltd. (Nanjing, China), and the cells were cultured in RPMI-1640 medium supplemented with 10% (*v*/*v*) fetal bovine serum, 100 IU/mL penicillin, and 100 μg/mL streptomycin, and incubated at 37 °C exposed to 5% CO_2_.

### 4.3. Exosome Extraction

A549 cells in logarithmic phase were exposed to SA1 for 48 h. The cells were divided into the SA1 group (final concentration 10 μM) and the control group. Then, the medium was harvested and the exosomes were obtained using Total Exosome Isolation Reagent (Thermo Fisher Scientific, Runcorn, Cheshire, UK) according to the manufacturer’s instruction. Briefly, the medium was centrifuged for 30 min at 2000× *g* and the supernatant was then centrifuged for 45 min at 12,000× *g* to remove the large vesicles. Thereafter, the supernatant was filtered through 0.45 μm filters, followed by centrifugation of the suspension for 70 min at 11,000× *g*. This centrifugation step was repeated to ensure purity. The final exosome pellet was resuspended and stored at −80 °C for future experiments. All steps were performed at 4 °C.

### 4.4. Assessment of Exosome Characteristics

The characterization of exosomes was performed according to our previous study [10]. Briefly, isolated exosomes were fixed in 2.5% glutaraldehyde, then stained, dehydrated, and embedded prior to observation under a JEM-1400 transmission electron microscope (JEOL, Tokyo, Japan). For protein expression analysis, exosomes from each group were resuspended in lysis buffer (4% SDS, 100 mM Hepes containing phosphatase inhibitor cocktail and 0.1% PMSF) and homogenized via sonication on ice for 10 min. The lysate was centrifuged at 25,000 *g* for 30 min to remove cellular debris. Total protein was collected from the supernatant, and its concentration was determined using a BCA assay kit (No. 23225, Thermo Fisher Scientific, Waltham, MA, USA). The expression of exosome protein markers was detected by Western blot analysis using antibodies against CD9 (1:1000) and CD63 (1:1000) and IgG-HRP (1:500). The chemiluminescent signals were detected by an ECL detection kit (KeyGen Biotech Co., Ltd., Nanjing, China). For the particle size analysis, the purified exosomes were suspended in PBS. Their particle size distribution and concentration were analyzed by the Flow NanoAnalyzer U30E (NanoFCM, Xiamen, China).

### 4.5. Animal Model Establishment and Anti-NSCLC Efficacy Study

BALB/c nude mice (4 weeks old) were bought from Shanghai Lingchang Biotech Ltd., Shanghai, China (SCXK-HU 2018-0003). The animal studies were authorized by the Laboratory Animal Welfare and Ethics Review Committee of KeyGen Biotech Co., Ltd., Jiangsu, China (IACUC-001-5). The animals were housed at 24 °C, with a relative humidity of 60–70%, under a 12 h light/dark cycle. All of them have a libitum access to food and water.

A549 cells in logarithmic phase were collected and adjusted to 1 × 10^7^/mL. A volume of 0.1 mL of the cell suspension was subcutaneously injected into the right forelimb of each mouse to establish the A549 xenograft model. Thereafter, the diameter of the transplanted tumors was checked regularly with a vernier caliper. When the diameter of tumors reached 100 mm^3^, the animals were randomly divided into four groups: model group (Model); control exosome group (Control exosome, 100 μg/mice); SA1 exosome group (SA1 exosome, 100 μg/mice); and Taxol group (Taxol, 8 mg/kg). Then, the mice were given exosome or drug intervention. The control exosome group was given exosome suspension twice a week by injection into the tumor, and the model group was given the same amount of normal saline twice a week with the same administration method and frequency. The Taxol group was administered Taxol by intraperitoneal injection twice a day. Throughout the experiment, the general condition and behavior of the mice were monitored dynamically and the weights of the mice were measured regularly. The lengths and widths of tumors were measured every 3 days, and the tumor volumes were calculated as follows: tumor volume = 0.5 × length × width^2^. At 28 days of treatment, all animals were sacrificed. Tumors were excised and weighed, and the tumor inhibition rate was calculated.

### 4.6. Sample Preparation for Proteomics Analysis

Proteins from each group (200 µg) were acetone-precipitated overnight and centrifuged at 15,000× *g* for 30 min to remove the acetone. The resulting pellet was resuspended in 50 µL of buffer (8 M urea, 50 mM Tris-HCl, pH 8.0, prepared in HPLC-grade water). An aliquot (2 μL) was taken from each sample for quantification using a BCA assay kit. The remaining proteins were reduced by 5 mmol/L dithiothreitol at 56 °C for 30 min and alkylated by 10 mmol/L IAA at room temperature for 30 min. The sample was then diluted with 50 mmol/L ammonium bicarbonate until the concentration of urea was lower than 1 M. After that, 4 μg of sequence grade trypsin was added to each protein sample at the mass ratio of 1:50 (trypsin: protein). The trypsin/protein mixture was incubated at 37 °C for 12 h and digestion was terminated by adding 1 μL of 100% trifluoroacetic acid. Samples were incubated for 5 min and centrifuged for 10 min at 25,000× *g* to obtain the peptide mixtures. The obtained peptide mixtures were then desalted by C18 STAGE tips, and lyophilized at 30 °C in a vacuum concentrator (Concentrator Plus, Eppendorf, Hamburg, Germany) and finally stored at −20 °C until further analysis.

### 4.7. Peptide Identification by LC-MS/MS

Proteome analysis was performed with a Fusion mass spectrometer (Thermo Fisher Scientific, Waltham, MA, USA). The dried peptide fractions were dissolved in 2% acetonitrile with 0.1% formic acid, and subsequently loaded on a C18 column (5 mm length, 100 µm inner diameter, Thermo Fisher Scientific, Bremen, Germany), followed by an analytical C18 column (250 mm length, 75 µm inner diameter, Eksigent). Typically, the elution gradients (Phase A, water-0.1% formic acid, Phase B, acetonitrile-0.1% formic acid) changed in 78 min at a flow rate of 300 nL/min (the gradient B ramped from 6% at 0 min to 10% at 8 min, 30% at 58 min, 40% at 70 min, and 95% at 78 min, respectively).

The mass spectrometer was operated in data-dependent acquisition mode to switch between Orbitrap-MS and ion trap acquisition automatically. An electrospray voltage of 2.0 kV versus the inlet of the mass spectrometer was used. Surveys of full-scan MS spectra (from *m*/*z* 300 to 1400) were acquired in the Orbitrap with a resolution of 120,000. Target ions already selected for MS/MS were dynamically excluded for 18 s and the minimum intensity was 5000.

### 4.8. Data Processing and Functional Analysis of DEPs

Protein quantification and label-free quantitative normalization of the MS/MS data were performed using MaxQuant (version 1.6.0.16, Max Planck Institute of Biochemistry, Planegg, Germany. https://www.maxquant.org/maxquant/, accessed on 2 December 2019). The andromeda configuration incorporated in the MaxQuant software was used to correlate MS/MS data against the Uniprot-Human database. Precursor mass tolerance was set to 20 ppm in the primary analysis and 4.5 ppm in the full search. Fragment mass tolerance was set to 0.5 Da. Search parameters were of strict trypsin specificity, allowing up to two missed cleavage sites. Oxidation of methionines residues (+15.995) and acetylation of protein N-terminal (+42.011) were set as variable modifications with carboxyamidomethylation (+57.021) being the fixed modification. False discovery rate was set to 0.01 for both peptides and proteins. Peptides with a minimum length of seven amino acids were considered for identification and proteins were only considered identified when observed in three replicates of one sample group.

Protein groups identified only by peptides with modified sites, contaminant matches, and matches to the reverse database were removed. Protein abundance was calculated on the basis of the normalized spectral protein intensity. The unpaired Student’s t-tests were used to compare the two groups. Only proteins with a probability for significant protein abundance changes with a *p* < 0.05 were used for fold change visualization. Hierarchical clustering calculations were performed by Multiple Experiment Viewer (https://sourceforge.net/projects/mev-tm4/files/mev-tm4/, accessed on 5 January 2020). GO term enrichment analyses for biological process, molecular function, and cellular component were conducted to identify potential mechanisms and processes that warranted further analysis. KEGG (https://www.kegg.jp/, accessed on 5 January 2020) and ingenuity pathway analysis (https://digitalinsights.qiagen.com/products-overview/discovery-insights-portfolio/analysis-and-visualization/qiagen-ipa/, accessed on 5 January 2020) were used for the pathway enrichment.

### 4.9. Bioinformatics Analysis of Database

The bioinformatics analysis of DEPs in lung cancer patients was performed using Kaplan–Meier Plotter database (https://kmplot.com/analysis/index.php?p=service&cancer=lung, accessed on 21 July 2025). The parameters for MFGM were Affy ID: 210605_s_at; split patient by: auto select best cutoff; survival: OS. The parameters for ANGL4 were Affy ID: 221009_s_at; split patient by: median; survival: OS. The parameters for GCN1 were Affy ID: 212139_at; split patient by: auto select best cutoff; survival: OS. The parameters for MUC5B were Affy ID: 222268_x_at; split patient by: auto select best cutoff; survival: OS. The parameters for APOB were Affy ID: 223579_s_at; split patient by: auto select best cutoff; survival: OS. Other parameters were default to the system. Then the Draw Kaplan–Meier plot option was selected to obtain the results of multivariate analysis.

### 4.10. Western Blot Analysis

Western blot was performed to verify the expression of targeted proteins. The extraction and quantification of proteins were completely identical with that used in label-free quantitative. Then, the quantified proteins (20 μg) from A549 and ADEs were separated by 5% SDS-polyacrylamide gel electrophoresis and transferred to a PVDF membrane (Mehler, Hückelhoven, Germany). Membranes were then blocked by 5% nonfat dry milk in TBST for 1.5 h at 25 °C. The band was washed with TBST and then incubated overnight with primary antibodies. The primary antibodies and concentrations were as follows: anti-MUC5B (1:1000), anti-MFGM monoclonal antibody (1:5000), anti-ANGL4 (1:1000), anti-GCN1 (1:3000), and anti-APOB (1:1000). GADPH in A549 cells was used as the internal reference. The total protein concentration in exosomes was used as the loading control. After being washed with TBST buffer, the membranes were incubated with a secondary antibody against rabbit IgG (1:2000) for 2 h at 25 °C. The membranes were subsequently washed with TBST and visualized using an ECL reagent.

### 4.11. Statistical Analysis

The results were provided as mean ± SD. Differences between groups were assessed through Student’s t-test or one-way ANOVA by SPSS 17.0 (IBM Corp., Armonk, NY, USA). *p* < 0.05 was assumed as statistically significant.

## 5. Conclusions

In summary, SA1 showed a notable anti-NSCLC property by regulating exosome function and altering the expression of associated proteins. Five potential targets (APOB, GCN1, MUC5B, MFGM and ANGL4) together with two key pathways (ribosome and spliceosome) may contribute to its efficacy and warrant further investigation. The study provides novel insights into the anti-NSCLC mechanism of SA1 through proteomic profiling and highlights its potential as a promising therapeutic candidate for lung cancer treatment.

## Figures and Tables

**Table 1:** Particle size distribution and the concentration of exosomes.

Groups	Particle Number	Size ($\overset{-}{x}$ ± SD, nm)	Peak Size (nm)	Concentration (/mL)
Control exosome	3076	78.25 ± 16.35	74.25	8.78 × 10^8^
SA1 exosome	6115	75.90 ± 14.14	72.75	3.34 × 10^9^

**Table 2:** Tumor inhibition rates of different treatment groups in A549 xenografts ($\overset{-}{x}$ ± SD, *n* = 8).

Groups	Tumor Inhibition Rates (%)
Model	/
Control exosome	15.94 ± 8.85 **
SA1 exosome	70.48 ± 5.41 **
Taxol	65.21 ± 6.28 **

** *p* < 0.01, compared with model group.

**Table 3:** The expression of DEPs in A549 cells vs. SA1-treated A549 cells (top 10).

ID	Entry Name	Ratio (SA1/A549)	LogRatio	*p* Value	Trend
P04114	APOB_HUMAN	2925.5951	11.5145	0.0014	up
P04808	REL1_HUMAN	892.2453	9.8013	0.0000	up
O43572	AKA10_HUMAN	521.9883	9.0279	0.0001	up
Q9HBL7	PLRKT_HUMAN	486.5595	8.9265	0.0019	up
P23229	ITA6_HUMAN	465.4387	8.8624	0.0020	up
P09012	SNRPA_HUMAN	0.0015	−9.3498	0.0001	down
P16403	H12_HUMAN	0.0015	−9.3786	0.0002	down
P37837	TALDO_HUMAN	0.0013	−9.5557	0.0001	down
P27695	APEX1_HUMAN	0.0012	−9.6502	0.0092	down
Q9HC84	MUC5B_HUMAN	0.0012	−9.7011	0.0011	down

**Table 4:** The expression of DEPs in exosomes from A549 cells vs. SA1-treated A549 cells (top 10).

ID	Entry Name	Ratio (SA1/A549)	LogRatio	*p* Value	Trend
Q92616	GCN1_HUMAN	2409.1026	11.2343	0.0000	up
P41252	SYIC_HUMAN	2112.1537	11.0445	0.0003	up
P14868	SYDC_HUMAN	1431.1157	10.4829	0.0000	up
P33993	MCM7_HUMAN	1188.8187	10.2153	0.0000	up
Q13085	ACACA_HUMAN	1094.3648	10.0959	0.0003	up
P00450	CERU_HUMAN	0.0001	−13.5016	0.0383	down
P17936	IBP3_HUMAN	0.0001	−13.5388	0.0139	down
Q13103	SPP24_HUMAN	0.0001	−13.8561	0.0109	down
Q9BY76	ANGL4_HUMAN	0.0001	−14.1495	0.0012	down
Q08431	MFGM_HUMAN	0.0000	−14.8569	0.0030	down

## Data Availability

The original contributions presented in this study are included in the article. Further inquiries can be directed to the corresponding author.

## Abbreviations

The following abbreviations are used in this manuscript:

NSCLC	Non-small cell lung cancer
TSGS	Total steroidal glycoalkaloids from *S. lyratum*
SA1	Solalyraine A1
ADEs	A549-derived exosomes
TDEs	Tumor-derived exosomes
DEPs	Differentially expressed proteins
GO	Gene ontology
KEGG	Kyoto encyclopedia of genes and genomes
MUC5B	Mucin-5B
APOB	Apolipoprotein B
MFGM	Lactadherin
ANGL4	Angiopoietin-related protein 4
GCN1	Stalled ribosome sensor GCN1
